# A meta-analysis on the efficacy of endoscopic ultrasonography for treatment of pancreatic cancer

**DOI:** 10.1016/j.clinsp.2024.100348

**Published:** 2024-03-28

**Authors:** Min Xuan, Na Li, Chunyan Wu

**Affiliations:** Department of Ultrasound, The Affiliated Wuxi People's Hospital of Nanjing Medical University, Wuxi People's Hospital, Wuxi Medical Center, Nanjing Medical University, Jiangsu Province, China

**Keywords:** Endoscopic ultrasonography (EUS), Pancreatic cancer, EUS-guided radiofrequency ablation, Randomized controlled trials, Meta-analysis

## Abstract

**Objective:**

This study aimed to systematically evaluate the efficacy and safety of Endoscopic Ultrasonography (EUS) for the treatment of pancreatic cancer.

**Methods:**

The PubMed, Embase, Web of Science, and Google Scholar databases were searched from the inception of the databases to June 2022. RevMan 5.3.0 software was utilized for data analysis. In total, 13 self-descriptive studies, which enrolled 382 patients, were finally included.

**Results:**

It was revealed that EUS for the treatment of pancreatic cancer exhibited a lower incidence of adverse reactions (Relative Risk Ration [RR = 0.23], 95 % Confidence interval [95 % CI 0.23–0.23]), a higher success rate (RR = 0.90, 95 % CI 0.90–0.90), and a low failure rate (RR = 0.06, 95 % CI 0.06–0.06). Moreover, EUS-guided Celiac Plexus Neurolysis (EUS-CPN) not only significantly relieved pancreatic cancer patients’ pain (RR = 0.83, 95 % CI 0.83–0.83), but also significantly eliminated pain in some patients (RR = 0.09, 95 % CI 0.09–0.09). The effects of EUS on pancreatic cancer treatment were satisfactory, and few adverse reactions were found.

**Conclusion:**

Owing to the restricted sample size in this meta-analysis, primarily consisting of descriptive studies, it was imperative to conduct more rigorously designed, multi-center, long-term follow-up, larger sample, and Randomized Controlled Trials (RCTs) to validate the findings.

## Highlights

• EUS-guided treatment for pancreatic cancer had fewer adverse reactions and a higher success rate.
• The direct injection of absolute ethanol into the abdominal ganglion exerted the best pain relief effects.
• US-guided Celiac Plexus Neurolysis (EUS-CPN) relieved and eliminated pain significantly.

## Introduction

Pancreatic cancer is a highly aggressive malignant tumor and is distinct from other malignant tumors in the gastrointestinal tract. Tumors are anatomically located in the retroperitoneum; however, comprehensive pathology and diagnoses can pose challenges. Moreover, tumors mainly exhibit unfavorable treatment outcomes. Approximately 50 % of patients with pancreatic cancer may suffer from metastasized tumors at diagnosis, while 30 % of patients with locally advanced disease cannot undergo surgery.^1^^,^^2^ Therefore, supportive care, palliative chemoradiotherapy, and palliative surgery are important treatment strategies.

Endoscopic Ultrasonography (EUS) encompasses a high-frequency ultrasound probe that is placed at the front end of an endoscope, scanning the gastrointestinal wall and surrounding adjacent organs, tissues, and suspicious lesions. EUS provides direct endoscopic and ultrasound imaging, which maximizes access to lesion sites and avoids air interference, thereby minimizing damage to surrounding organs and tissues. EUS also improves diagnostic accuracy, and it is widely utilized for diagnosing pancreatic diseases.^3^ In recent years, the ongoing advancement of EUS technology has resulted in notable technological progress, evolving from a diagnostic tool to a minimally invasive interventional treatment method.^4^, ^5^, ^6^ At present, EUS is a notable diagnostic method for pancreatic cancer in China.

Pancreatic cancer is prone to local invasion of the retroperitoneal plexus. EUS-guided Celiac Plexus Neurolysis (EUS-CPN) can relieve pain by injecting therapeutic drugs into the celiac ganglion. The EUS-CPN typically uses absolute ethanol or phenol as a solvent. Minaga et al.^7^ found that the efficacy rate of EUS-CPN in reducing pain was 73.5 %–90.2 %. Moreover, the efficacy may last for approximately 8 weeks, and analgesic effects are satisfactory.

EUS-guided Radiofrequency Ablation (EUS-guided RFA)^8^ is utilized to treat local tumors in a minimally invasive and safe manner. EUS-RFA may relieve pain symptoms caused by tumors. Scopelliti et al.^9^ assessed the feasibility and safety of EUS-guided RFA for unresectable Pancreatic Ductal Adenocarcinoma (PDAC), and it was indicated that the technique was successful in all cases, with no major adverse events recorded. In all cases, a delineated hypodense ablated area within the tumor was observed using the 30-day Computed Tomography (CT) scan. Thus, the authors suggested that EUS-guided RFA was a feasible and safe, minimally invasive procedure for patients with unresectable PDAC.

Pancreatic cancer is characterized by a low surgical resection rate and a poor prognosis.^10^ The treatment of unresectable Locally Advanced Pancreatic Cancer (LAPC) remains highly controversial,^11^ with the current standard of care limited to chemotherapy and/or radiotherapy.^12^ EUS-guided radioactive seed implantation can be applied to treat LAPC or recurrent pancreatic cancer. Notably, EUS enables precise navigation to avoid crucial structures, including blood vessels and pancreatic ducts. The spatial distribution of radioactive seed is more uniform, and the number of EUS-induced complications is limited. Bhutani et al.^12^ utilized EUS-guided isotope P_32_ microparticle brachytherapy, and combined it with gemcitabine to treat LAPC. The results indicated that all 9 patients were successfully treated with the mentioned therapeutic regimen, and there was no serious adverse reaction related to surgery, suggesting that EUS-guided brachytherapy appeared technically feasible.

To date, several clinical studies have demonstrated that the therapeutic efficacy of EUS for pancreatic cancer is satisfactory.^13^, ^14^, ^15^, ^16^ However, at early research stages, due to small sample sizes, no meaningful references for clinical practice have been presented, and no comprehensive meta-analysis was carried out. Therefore, this meta-analysis was conducted to systematically evaluate the efficacy and safety of EUS for the treatment of pancreatic cancer to provide evidence-based information for clinical practice.

## Methods

### Search strategy

The PubMed, Embase, Web of Science, and Google Scholar databases were searched for retrieving the relevant articles. The search timeline was from the inception of the databases to June 2022, and only English-published articles were retrieved. The MeSH terms included “endoscopy”, “endoscopic ultrasonography”, “pancreatic cancer”, “EUS”, and “treatment”. A combination of key and subject words was employed to conduct a comprehensive search.

### Inclusion criteria

(1) The publication language was limited to English; (2) The trial design adhered to scientific principles and was deemed reasonable, with consistent statistical methods across studies; (3) Study subjects were pancreatic cancer patients; (4) The outcome indicators included: (a) Success rate; (b) Pain relief status; (c) Occurrence of adverse reactions; and (d) Complete pain relief status; (5) Patients were self-aware, and patients and their families voluntarily participated in the research; (6) All participants or their guardians signed informed consent forms, and the research was approved by a local ethics committee.

### Exclusion criteria

(1) Reviews, systematic reviews, case analyses, and meta-analyses; (2) Duplicate publications; and (3) Data extraction difficulties.

### Study screening

All studies were independently searched, screened, and reviewed by two researchers. Any discrepancies encountered during the review process were eliminated through discussions or expert judgment. Data extracted from studies mainly included: (1) First author's name and publication date; (2) Sample size, average age, male/female, type of pancreatic cancer; intervention measures, success status, failure status, pain relief, no pain status and adverse reaction status; (3) Specific interventional measures; (4) Outcome indicators.

### Statistical analysis

RevMan 5.3.0 software was used to analyze data extracted from selected studies. If outcome variables were dichotomous, the relative Risk Ratio (RR) was calculated. If outcomes were continuous variables, the Mean Difference (MD) was calculated. Both measures used 95 % Confidence Intervals (95 % CIs). The I^2^ statistic was calculated to assess heterogeneity among studies. The χ2 test was utilized for statistical testing. In cases where there was no significant heterogeneity among the results of each study (I^2^ <50 % and p > 0.1), a fixed-effects model was utilized for meta-analysis. If there was statistical heterogeneity among the results of each study (I^2^ ≥ 50 %, or p ≤ 0.1), a random-effects model was utilized for meta-analysis after excluding the influence of obvious clinical heterogeneity. For studies exhibiting substantial clinical heterogeneity, subgroup, sensitivity, or descriptive analyses were conducted. The level of significance was set at α = 0.05.

## Results

### Retrieved articles

In total, 1596 articles were initially retrieved, of which 1048 duplicate studies, reviews, and abstracts were excluded. After reading titles and abstracts of 548 articles, 477 articles were eliminated, and 71 articles remained. The full text of 71 articles was subsequently read, and 59 articles that did not meet study requirements were removed. Finally, 13 articles were analyzed.

### Basic characteristics of the eligible studies

A total of 13 articles were included in the meta-analysis.^17^, ^18^, ^19^, ^20^, ^21^, ^22^, ^23^, ^24^, ^25^, ^26^, ^27^, ^28^, ^29^ Their basic characteristics are presented in Table 1.

**Table 1:** Basic characteristics of the selected studies.

Author(s), publication time	Sample size (cases)	Male/Female	Average age (years)	Disease type	Intervention measures	Success (cases)	Failure (cases)	Pain relief (cases)	No pain (cases)	Adverse events	Outcome indicators
LeBlanc et al., 2013^17^	10	5/5	66±14	Pancreatic cancer-related pain	10 mL alcohol during EUS-CPN	‒	‒	8	‒		3
Gunaratnam et al., 2001^18^	58	32/26	67.1±9.3	Pancreatic cancer	EUS-CPN	‒	‒	45	‒	Procedure-related transient abdominal pain (5 patients)	3
LeBlanc et al., 2011^19^	50	24/26	63	Pancreatic cancer related pain	alcohol given as 1 vs. 2 injections during EUS-CPN	‒	‒	37	4	There were no long-term complications	3, 4
Wiechowska-Kozłowska et al., 2012^20^	29	‒	‒	Advanced and unresectable pancreatic cancer	EUS-CPN	‒	‒	25	4	Hypotonia (1 patient), severe pain immediately post-procedure (2 patients), short episodes of diarrhea (3 patients)	3, 4, 5
Hao et al., 2014^21^	41	24/17		Unresectable pancreatic cancer	EUS-CPN	‒	‒	23	4	Transient hypotension (2 patients)	3, 4, 5
Sanders et al., 2010^22^	51	29/22	73	Either locally advanced or recurrent pancreatic cancer	EUS-SBRT	46	4	‒	‒	Mild pancreatitis (1 patient), simultaneous placement of fiducials and celiac plexus neurolysis for intractable abdominal pain (1 patient)	1, 2, 5
Sun et al., 2012^23^	8	3/5	69	Advanced pancreatic cancer	(EUS-ICR)	4	‒	‒	‒	‒	1
Figueiredo et al., 2021^24^	37	20/17	60	Borderline resectable/locally advanced pancreatic ductal adenocarcinoma	EUS-SBRT	34	3	‒	‒	Three patients (8 %) had adverse events (fever, mild acute pancreatitis, and biliary stent migration)"	1, 2, 5
Park et al., 2010^25^	57	29/28	67 ± 12	Locally advanced unresectable pancreatic adenocarcinoma	EUS-guided insertion of gold fiducials	50	3	‒	‒	3 patients	1, 2, 5
Sun et al., 2006^26^	15	8/7	61	Locally advanced pancreatic cancer	EUS-guided interstitial brachytherapy	12	‒	‒	‒	Pancreatitis and pseudocyst formation (3 patients); grade III hematologic toxicity (3 patients)	1, 5
Bang et al., 2019^27^	12	5/7	62.8±13.7	Pancreatic cancer	EUS-RFA	12	‒	‒	‒	5 patients	5
Crinò et al., 2018^28^	8	3 (5)	67.75	Pancreatic adenocarcinoma	EUS-RFA was performed using 18-gauge internally cooled electrode with a 5- or 10-mm exposed tip	8	‒	‒	‒	Mild post-procedural abdominal pain (3 patients)	5
Song et al., 2016^29^	6	1/5	62	Unresectable pancreatic cancer	EUS-RFA	6	‒	‒	‒	Mild abdominal pain (2 patients)	5

Note: EUS, Endoscopic Ultrasound; CPN, Celiac Plexus Neurolysis; SBRT, Stereotactic Body Radiotherapy; ICR, Interstitial Chemoradiation (EUS-ICR); RFA, Radiofrequency Ablation; Outcome Indicators: 1 EUS-guided related surgery was successful; 2 EUS-guided related surgery was failure; 3 Pain relief; 4 There was no pain after EUS-guided related surgery; 5 Adverse events.

## Meta-analysis results

### Incidence of adverse reactions

**Fig. 1:**
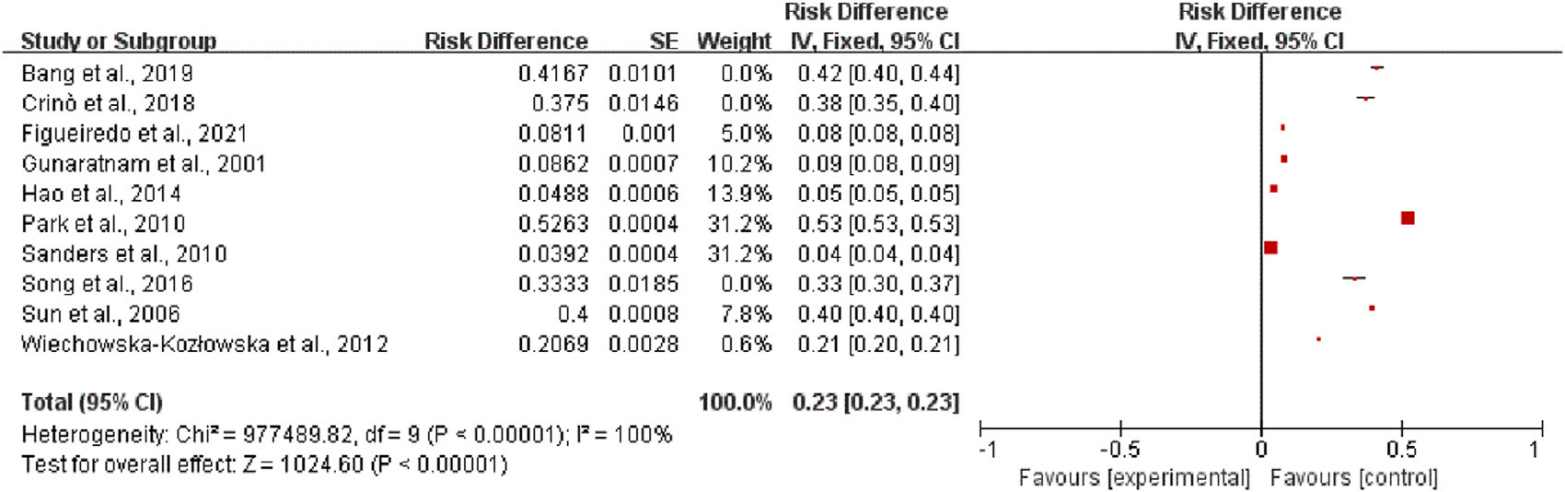
Meta-analysis results indicating the incidence of adverse reactions.

Among 13 studies, the incidence of adverse reactions was assessed in 10 studies.^18^^,^^20^, ^21^, ^22^^,^^24^, ^25^, ^26^, ^27^, ^28^, ^29^ to explore the efficacy of EUS for the treatment of pancreatic cancer. These studies were self-descriptive in nature. It was found that 26 patients in 3 studies were successfully treated with EUS-guided RFA for pancreatic cancer. The heterogeneity test result for the incidence of adverse reactions was I^2^=100 % (*p* < 0.00001). Thus, the incidence of adverse reactions during pancreatic cancer treatment with EUS was low (RR=0.23, 95 % CI 0.23–0.23) (Fig. 1).

### Success rate

**Fig. 2:**
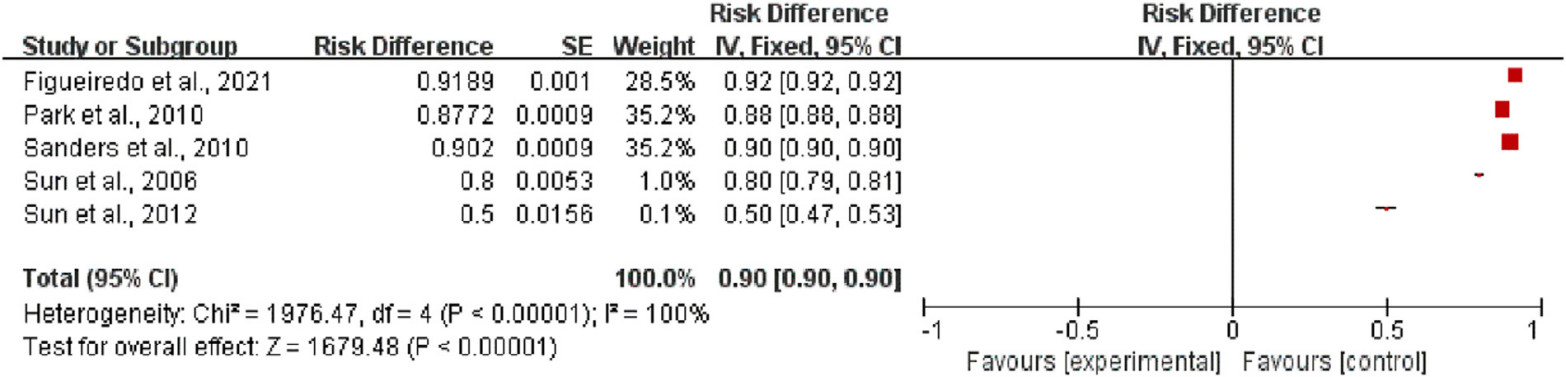
Meta-analysis results indicating the success rate.

Among the 13 studies, the success rate was assessed in 5 studies^22^, ^23^, ^24^, ^25^, ^26^ to examine the effects of EUS on the treatment of pancreatic cancer. The heterogeneity test result for the success rate was I^2^ = 100 % (*p <* 0.00001). Thus, EUS exhibited a high success rate for the treatment of pancreatic cancer (RR = 0.90, 95 % CI 0.90–0.90) (Fig. 2).

### Failure rate

**Fig. 3:**
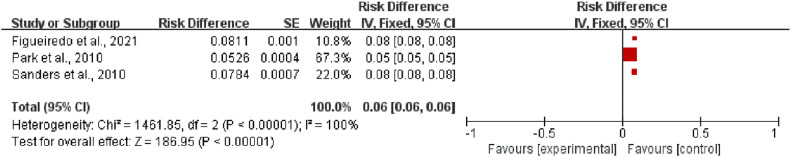
Meta-analysis results indicating the failure rate.

Among the 13 studies, the success rate was assessed in 3 studies^22^^,^^24^^,^^25^ to explore the effects of EUS on the treatment of pancreatic cancer. The heterogeneity test result for the failure rate was I^2^=100 % (*p <* 0.00001). Thus, the EUS exhibited a low failure rate for the treatment of pancreatic cancer (RR = 0.06, 95 % CI 0.06–0.06) (Fig. 3).

### The incidence of pain relief after EUS-CPN treatment

**Fig. 4:**
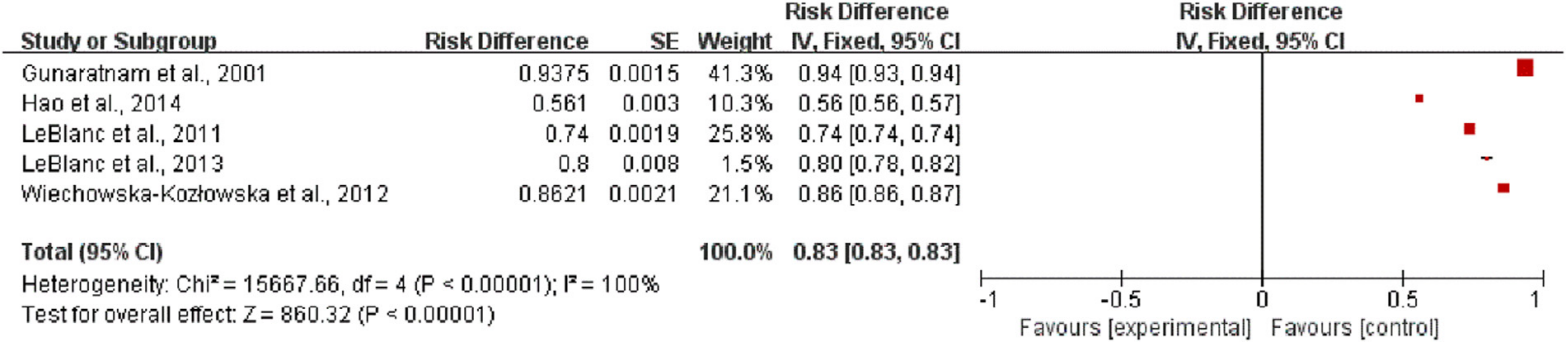
Meta-analysis results indicating the incidence of pain relief after EUS-CPN.

Among the 13 studies, the incidence of post-treatment pain relief was assessed in 5 studies^17^, ^18^, ^19^, ^20^, ^21^ to examine the effects of EUS-CPN on pain relief in patients with pancreatic cancer. The heterogeneity test result for the incidence of post-treatment pain relief was I^2^ = 100 % (*p <* 0.00001). Thus, EUS-CPN significantly relieved pain in patients with pancreatic cancer (RR=0.83, 95 % CI 0.83–0.83) (Fig. 4).

### The incidence of no pain relief after EUS-CPN treatment

**Fig. 5:**
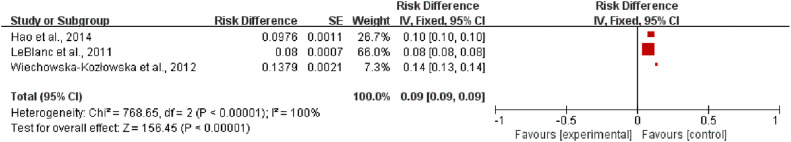
Meta-analysis indicating the incidence of no pain relief after EUS-CPN.

Among the 13 studies, the incidence of no post-treatment pain relief was assessed in 3 studies 19, 20, 21 to explore the effects of EUS-CPN on pain in patients with pancreatic cancer. The heterogeneity test result for the incidence of no post-treatment pain relief was I^2^ = 100 % (*p <* 0.00001). Therefore, EUS-CPN significantly eliminated pain in pancreatic cancer patients (RR = 0.09, 95 % CI 0.09–0.09) (Fig. 5).

## Discussion

Pancreatic cancer is a highly malignant tumor of the digestive system, and it primarily originates from atypical hyperplasia of the pancreatic ductal epithelium. The majority of patients have no obvious symptoms at early disease stages; however, they mainly progress to locally advanced stage at the time of diagnosis. Combined with distant metastasis, no radical surgery can be performed.^30^ At present, most patients with unresectable advanced pancreatic cancer are treated with gemcitabine-based chemotherapy combined with *in vitro* and *in vivo* radiotherapy. However, pancreatic tumors are highly active, and early metastasis and recurrence may occur even after treatment. Thus, the treatment of pancreatic cancer is a clinical challenge.

EUS can be utilized for the short-range, high-resolution, real-time imaging of the pancreas and surrounding structures. EUS-guided puncture provides accurate positioning, less trauma, and short puncture distances. In recent years, EUS has not only been utilized to diagnose pancreatic lesions, but also it has become a new interventional method for the treatment of pancreatic cancer.^31^

Using EUS-guided RFA, the ablation electrode can be accurately inserted into the pancreatic lesion under real-time ultrasound guidance, permitting accurate tumor ablation. Currently, EUS-RFA is utilized for a variety of pancreatic diseases, including pancreatic cystic tumors and pancreatic neuroendocrine tumors.^32^^,^^33^

Few effective therapies are available to control locally advanced or metastatic pancreatic cancer. External irradiation therapy is insensitive and associated with systemic side effects, although it can relieve pain in up to 50 %–85 % of patients.^34^ The widespread utilization of EUS and therapeutic EUS has enabled the feasibility of radioactive seed implantation into solid tumors via EUS. It is generally accepted that EUS-guided puncture facilitates accurate positioning, mild injury, and shorter puncture distances versus conventional ultrasound approaches. Radioactive seed implantation is the implantation of radioactive seeds according to the size and shape of the tumor under the positioning and guidance of imaging equipment. The implantation site is tissue within the tumor or infiltrated by the tumor. The surgical intervention represents a variant of brachytherapy, emitting sustained, short-range localized gamma rays from minuscule radioactive sources. The gamma rays produced by the radioactive particles can destroy the tumor tissue to the greatest extent, and they have little or no effect on the normal tissue. Its application to pancreatic cancer research is more extensive in China than overseas. The safety of EUS-guided interstitial implantation of radioactive seeds has been proven in animals^34^ and humans.^35^ In 2005, Sun et al.^35^ implanted isotope I_125_ particles into the pancreatic tissue of experimental pigs using EUS, and they confirmed the safety and feasibility of the method. In a subsequent clinical trial,^26^ these authors enrolled 15 patients with advanced pancreatic cancer (8 patients with stage III and 7 patients with stage IV). Each patient underwent the implantation of 22 inoperable seeds. No patient received postoperative chemotherapy. The effective treatment rate was 80 % (4 cases with partial remission, 3 with slight improvement, and 5 with no deterioration). Among the four partial remission patients, a reduction in tumor diameter exceeding 50 % was found within the initial 3 months, with remission lasting for 4‒5 months. Additionally, the quality life in 5 patients was improved within 1 month by comparing preoperative and postoperative Visual Analogue Scale (VAS) pain and Karnofsky Performance Status (KPS) physical strength scores. Peretz et al.^36^ implanted isotope I_125_ seeds visibly during surgery to treat 98 patients with pancreatic cancer, and achieved a response rate of 45 % and a pain relief rate of 65 %. For chemotherapy, meta-data from a previous Randomized Controlled Trial (RCT)^37^ showed that median 6-month and 1-year survival rates were 51 % (20.3 ± 81.3 %) and 21 % (11.0 ± 37.2 %) in the gemcitabine alone group, and 58 % (31.1 ± 68 %) and 23.3 % (6.3 ± 38.5 %) in the gemcitabine plus EUS group, respectively. Wang et al.^38^ performed an EUS-guided isotope I_125_ seed implantation study in the celiac ganglion to treat intractable abdominal pain in 23 patients with inoperable pancreatic cancer. An average of four isotope I_125_ seeds were implanted into the celiac ganglia of each patient, and all patients received gemcitabine chemotherapy postoperatively. The study reported no significant changes in VAS pain scores, and morphine-type analgesic usage by patients on the day and the first week after surgery. However, 19 (82.6 %) patients experienced pain relief 2-weeks after surgery. At the postoperative 5-month mark, 50 % of patients still experienced partial pain relief. Bhutani et al.^39^ employed EUS-guided isotope P_32_ seed implantation to treat advanced pancreatic cancer. They completed the implantation operation on the 4th week of the first stage of treatment with nab-paclitaxel combined with gemcitabine. Implanted particle activity was 12.3 M Bq. No obvious postoperative adverse reaction was found in 9 patients, and they continued treatment with chemotherapy. In the 16th week after the surgery, CT scan revealed that the average tumor volume was reduced by 58 % (23.2 mL reduced to 9.7 mL). At the 22nd week, the patient's abdominal pain symptoms were completely relieved, and serum CA19-9 level decreased from 635 to 25 U/mL. Thus, EUS-guided^32^ P seed implantation was effective for the patients with advanced pancreatic cancer.

Stereotactic Body Radiation Therapy (SBRT) uses advanced imaging techniques to confirm lesion location before and during treatment to provide high-dose radiation to accurately target pancreatic tumors.

EUS-CPN is a relatively new approach, and it effectively reduces the risk of bleeding and non-surgical site damage to patients.^40^^,^^41^ The operation is entirely performed under color Doppler guidance, effectively avoiding blood vessels during the puncture process. The needle track is displayed by ultrasound, improving puncture accuracy. Pain is the main symptom in patients with advanced pancreatic cancer. Traditional treatment mainly uses opioids for pain relief. However, long-term high-dose administration of opioids can produce tolerance, and patients may be addicted to opioids after long-term usage.^42^

CPN for the treatment of advanced pancreatic tumor pain has been reported and applied by several clinical centers, and a number of studies have confirmed that this method is effective for the pain relief of pancreatic cancer patients.^43^ CPN is mostly performed under the guidance of CT and B-mode ultrasound, which is prone to damage to the patient's blood vessels and organs. With the development of interventional endoscopic ultrasonography, EUS-guided CPN can effectively reduce the risk of bleeding and non-surgical site damage to patients.^44^^,^^45^ The present meta-analysis revealed a lower incidence rate of adverse reactions (RR = 0.21, 95 % CI 0.21–0.22), a higher success rate (RR = 0.90, 95 % CI 0.90–0.90), and a lower failure rate (RR = 0.06, 95 % CI 0.06–0.06) resulting from the application of EUS for treatment of pancreatic cancer.

Pancreatic cancer pain management is a clinical challenge as patients mainly require large analgesic doses for pain relief. In some instances, the addictive nature of drugs, adverse reactions, and poor pain relief effects make it difficult for patients to tolerate these drugs. Therefore, the utilization of EUS-CPN in clinical practice may reduce analgesic doses. Vranken et al.^46^ performed pathological examinations on two cadaveric patients with pancreatic cancer who received anhydrous ethanol injection into the abdominal ganglion. They found that the nerve capsule was partially coagulated and necrotic. Hyaline degeneration appeared in a small number of nerve fibers. However, most of the neurons were intact. They hypothesized that CPN-based injection of chemical nerve-damaging agents did not cause permanent neuronal necrosis. Thus, they assumed that CPN did not completely eliminate pain, while it only temporarily relieved it. However, Ascance et al.^47^ conducted a retrospective analysis of 64 patients with pancreatic cancer, and they compared 40 patients who received direct intraganglionic injection and 24 patients who received bilateral celiac trunk injection. After 4 weeks, 26 (65 %) patients who received direct injection into the celiac ganglia experienced pain relief, while only 6 (25 %) patients who received bilateral injection into the celiac trunk experienced effective pain relief. These results demonstrated that the direct injection of absolute ethanol into the abdominal ganglion exerted the best pain relief effects. In the present meta-analysis, 138 pancreatic cancer patients experienced pain relief after EUS-CPN treatment. The results revealed that EUS-CPN not only significantly relieved pain in pancreatic cancer patients (RR=0.83, 95 % CI 0.83–0.83), but also pain was eliminated in 12 pancreatic cancer patients after EUS-CPN treatment. However, the results indicated that EUS-CPN could significantly eliminate pain in pancreatic cancer patients (RR = 0.09, 95 % CI 0.09–0.09).

## Conclusions

This meta-analysis, accessible online, provided evidence for the efficacy of EUS for the treatment of pancreatic cancer based on non-randomized studies. Robust evidence for evaluating the comparative efficacy and safety of EUS for the treatment of pancreatic cancer in these studies is currently lacking. However, it is essential to avoid underestimating data from cohort studies and non-RCTs, as these studies can better realize clinical settings compared with RCTs. Moreover, RCTs may not always be feasible under certain clinical circumstances. Hence, a combination of RCTs, non-RCTs, and cohort studies is invaluable for obtaining more accurate outcomes in clinical practice.

## Ethics approval and informed consent

Not applicable.

## Availability of data and materials

All data generated or analyzed during this study are available from the corresponding author upon reasonable request.

## Authors’ contributions

Min Xuan: Investigation, Methodology, Data curation, Writing.

Na Li: Data curation.

Chunyan Wu: Project administration, review & editing.

All authors have read and approved the final manuscript.

## Conflicts of interest

The authors declare no conflicts of interest.

## Appendix Supplementary materials

Image, application 1
