# Supplementary figures and images for: A meta-analysis on the efficacy of endoscopic ultrasonography for treatment of pancreatic cancer
Source: Clinics (Sao Paulo). 2024 Mar 28;79:100348. doi: 10.1016/j.clinsp.2024.100348 (PMC10998044; doi:10.1016/j.clinsp.2024.100348)

**CLINICS-D-23-00219_ Supplementary Material**

PRISMA flowchart.


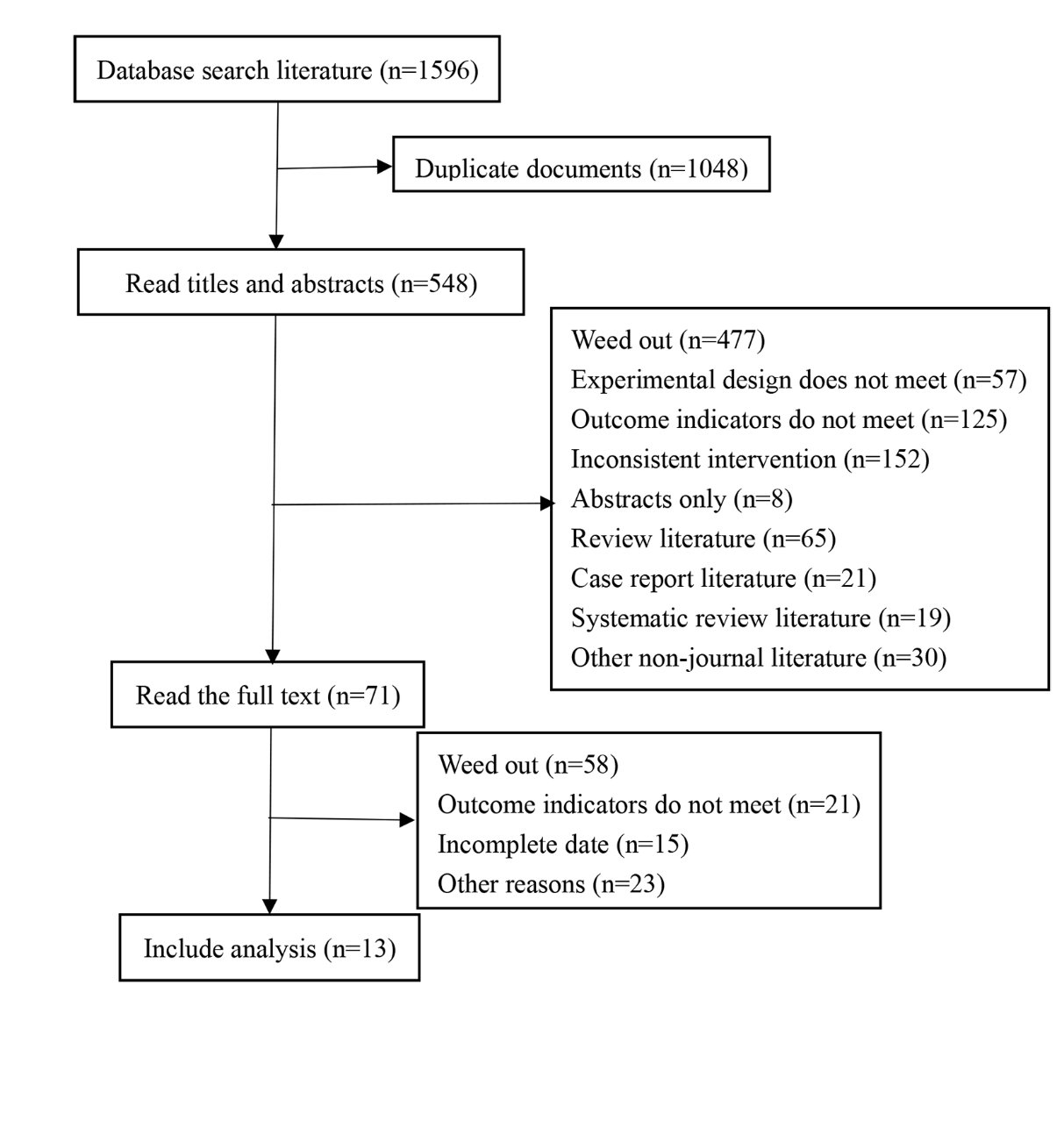

Supplement: Supplementary file 1 [file mmc1.docx]
